# List of Authors

**DOI:** 10.1093/eurpub/ckac132

**Published:** 2022-11-02

## A

Aadahl M   70

Abdelghyoum mahgoub A   90

Abdrakhmanova S   6, 7

Abdurrahmonova Z   6, 8

Abel T   43, 109

Abildina A   6

Abu-Omar K   6, 8, 27, 43, 61, 66, 101, 104, 107, 109

Acs P   96

Ács P   95

Adams L   73

Adrian B   96

Aguenaou H   33, 113

Agyapong-Badu S   36, 83, 84

Ahlen J   78

Ahrens W   6, 7

Aira T   57

Aitmurzaeva G   6

Alarslan G   105

Alfonso-Rosa RM   114

Allen K   47, 62, 65

Allesøe K   92

Altmeier D   27, 29

Amholt TT   81

Amiama J   49

Amort FM   107

Anastasi D   73

Andermo S   57

Andersson G   58

Andkjær S   105

Andreas H   46, 50, 86, 115

Andreasyan D   8

Angelo VFS   6

Antoine-Jonville S   71

Aranda-Balboa MJ   111

Arnaiz P   7, 73

Arvidsen J   105

Ashworth E   17

Astruc A   100

Asun S   71

Asurmendi U   49

Autrup SK   107

## B

Bälter K   38

Bøggild H   70

Börjesson M   88

Büsching G   72

Bache A   102

Backes L   42

Baddou I   33, 113

Bakalár P   90

Balboa MJA   32

Banchoff A   84

Barón-López FJ   45

Barbosa A   7, 73

Barnes A   16

Barnes L   116

Barranco-Ruiz Y   32, 111

Barrett E   36

Barros TVP   42

Bauman A   25, 38

Baumann M   93

Belander O   35

Bellis MA   41

Belton S   10, 16, 55

Bengoechea EG   23, 24, 35, 50, 71, 91

Benjaminse A   56

Benjeddou K   33, 113

Bentsen P   9, 10, 80

Berdzuli N   8

Bergh IH   6, 7

Berjaoui R   63

Bernal C   52, 56

Bertozzi N   87

Bezerra TA   54

Bhawra J   77

Bigot L   44, 118

Bilsborough H   82

Birdsall-Strong L   4, 5

Blackmore C   85

Blagus R   8

Blanchard J-C   100

Bloch P   70

Blom V   58

Blond M   120

Bocquier A   34

Boddy LM   17

Boen F   13, 14, 36

Bohouri H   71

Bois J   50, 52, 56, 68

Bojkowska I   31

Bollen J   34

Bolton N   40, 69

Borodulin K   13, 14

Boyle H   74

Boymatova K   6, 7

Brønd JC   9

Brandes M   8

Breda J   6, 7, 8

Briançon S   44, 53, 55

Brinduse LA   6, 7

Brito J   73

Broom D   37

Brown S   84

Brun P   72

Buck C   117

Bucksch J   31

Buli BG   38

Buoncristiano M   6, 7

Burnard MD   89

Busch V   81

Byrne E   59

## C

Caetano R   41

Calogiuri G   59

Campostrini S   98

Cardoso J   41

Carillo S   112

Carl J   27

Carlin A   15, 17

Carré F   113

Carreira B   99

Carretier J   74

Carron T   33

Carrozzi G   87, 98

Casey B   24

Castetbon K   111

Castro-Piñero J   39, 66

Ceciliani A   66

Cerini T   72

Česnaitienė V   83

Chaba L   47

Chalabaev A   120

Chalkley A   38, 52

Chan DK-C   94

Cheilleachair NN   72

Chemla R   103

Chenuel B   101

Chereches RM   61, 102

Cheryl W   73

Chillón P   32, 111

Chinapaw M   69

Christèle G   64, 113

Christensen JH   80

Christian H   44

Christiansen LB   31, 78, 109

Ciaran D   35

Ciccarelli M   73

Cillekens B   46, 50, 86, 115

Clark CCT   54

Clays E   46, 50, 61, 86, 115

Clayton D   71

Clifford A   35

Clifford L   17

Cloes M   54, 102, 103

Clynes J   34

Coeli C   41

Coenen P   46, 50, 86, 115

Collard D   69, 105

Collmann C   98

Colom A   45

Conde-Caveda J   39

Constable Fernandez C   76

Contoli B   87, 97, 98

Coppinger T   52

Corr M   16

Corrion K   94

Costa S   97

Covaliu B   102

Cowen C   4

Crispo A   8

Cristofori M   87

Crone D   40, 69, 71, 75

Cross R   34

Cruwys T   36

Csanyi T   8

Cucu A   6, 7

Čustonja Z   95

## D

Dóczi T   95

D'Argenzio A   98

Da S   103

Dakin M   53

Dalgas BW   81

Darmon N   34

David S-O   66

De Bon D   119

De Clerck I   58

De Cocker K   71

De Jong J   69, 70

De Luca AMC   87

d'Arripe-Longueville F   45, 47, 94, 115, 119

da Silva BS   42

de Hollander E   20, 21, 97

de Jager D   105

de Jong J   33

de Jonge M   55

de Koning J   34

de Oliveira JAG   41

del Pozo-Cruz B   114

del Pozo-Cruz J   114

den Braver NR   24

den Uil A   81

denBraver N   22

dos Santos DL   42

Degen J   73

Delagardelle C   102

Delrieu L   74

Demant Klinker C   70

Derigny T   80

Desnouck V   111

Digennaro S   73

Dippon L   27, 37, 107

Doczi T   96

Dodd-Reynolds C   48

Doherty LC   15, 17

Dolley D   73

Domokos B   31

Donnelly A   35, 91

Dornelles N   42

Dowd K   72, 82

Drozdova-Statkeviciene M   83

Du Randt R   73

Dubertrand L   50, 68

Dubois C   34

Dubuisson C   112

Duché P   112

Duclos M   113, 120

Duffey K   7

Duhair A   108

Duijvestijn M   97, 114

Dujeu M   111

Duleva V   6, 7

Dunca M   54

## E

Ek A   88

Ekberg J-E   62

Ekblom-Bak E   58, 87, 109

Ekblom Ö   78, 88, 91

Ekblom B   87

Ekinci B   8

Ekström M   88

El Hamdouchi A   113

El Harchaoui I   113

El Kari K   113

El Menchawy I   113

El Ouadrhiri Y   112

Elinder LS   39

Elkrog-Hansen LM   78

Elkrog-Hansen L   51

Elmzibri M   33, 113

Elsborg P   9, 10

Em S   108

Erguder T   8

Ericson H   85

Eriksen MB   109

Erjavec IŠ   47, 106

Esteban-Cornejo I   39

Evans AB   80

Evans C   46

## F

Fabre N   50, 52, 56, 68, 71

Fairclough SJ   17

Falzon C   103

Fanian D   62, 65

Fateh-Moghadam P   87, 98

Faulkner M   15, 17

Febvey-Combes O   74

Federico B   73

Fedkina N   8

Fell T   38

Fernández M   32

Ferschl S   27, 43, 109

Fervers B   74

Figueiredo P   73

Fijałkowska A   7

Fijalkowska A   6

Filleul V   94

Fiona M   49

Fismen A-S   6, 47, 65

Fitó M   45

Fjørtoft I   62

Fonvig CE   79

Forberger S   22, 23, 24

Ford K   41

Foweather L   17

Fox K   36

Frahsa A   27

Franck N   54, 103

Fransen K   14, 36

Frei A   33, 72

Freidl A   108

Frisch A   102

Froberg K   62

Fromel K   51

Fuchs A   44

## G

Günther L   118

Gage R   96

Gahraman H   8

Gallagher AM   15, 17

Gallagher A   15

Gallagher S   35

Gallardo K   75

Gallardo O   75

Gallardo R   75

Gallo R   97

Gandrieau J   80

Garbarino J-M   41, 48

García-Solano M   6, 7

Garcia E   22

Gauthier A   118

Gautier C   63

Gavin K   18, 19

Geidl W   101, 104

Geidne S   18, 20, 85

Gelius P   6, 8, 22, 23, 24, 27, 28, 43, 49, 61, 66, 101, 104, 109

Gemmerich A-M   44

Gerber M   73

Ghebghoub Y   108

Giacomini M   42

Giroud P   33

Gluchowski A   82

Glynn L   35, 91

Gobis A   22, 23

Godinho CA   32

Godinho C   38

Grüne E   27

Grady C   50, 68, 71

Graf E   119

Grao-Cruces A   66

Greaves C   34

Großschädl L   12

Groenewoud S   93, 94

Groffik D   51

Gualtieri A   6, 7

Guillemin F   44, 55, 117

Guler M   33

Gumhold V   108

Gupta N   46, 50, 86, 89, 115

Gusi N   39

Gutiérrez-González E   6, 7

Gutter K   105

## H

Høyer-Kruse J   109

Hadžiomeragić AF   6

Haerens L   68

Hagströmer M   120

Hakonen H   98, 110

Halgand F   119

Hallman D   46, 50, 86, 89, 115

Hamdouchi AE   33

Hannigan A   91

Hansen AF   109

Harbron J   47, 65

Harchaoui IE   33

Harrington D   52

Harris M   42

Haslam C   36

Hassapidou M   6

Hassel H   27

Hawkes RA   84

Hayes G   91

Hayotte M   45, 115, 119

Heiden M   115

Heijnen E   49

Heinen MM   6

Heinonen O   57

Hejgaard T   6, 7

Helgadóttir B   78, 91

Hellénius M-L   57

Helleve A   47, 62, 65

Helsper N   27, 37, 107

Hemmingsson E   109

Hendricks G   47

Herbinet A   44

Herrador-Colmenero M   32

Hesketh KD   39

Hill D   78

Holler P   107

Holm Pisinger C   70

Holmberg E   111

Holmlund T   87

Holtermann A   89

Horvat M   82

Hue O   71

Huertas-Delgado FJ   32, 111

Hughes K   41

Hughes R   66

Huhtiniemi M   54, 77

Huiberts I   69

Hurter L   78

Husu P   22

Hyska J   6, 7

## I

Ibor E   71

Ionnaidu EM   6

Iserbyt P   14, 36

Issartel J   10

Iturrioz I   49

Ivanova K   118

## J

Jørgensen MB   7

Jørgensen TS   70

Jaakkola T   54, 77

Jago R   15, 17

Janky E   71

Janssen M   81

Januario L   115

Jepkema N   69, 70

Jespersen JF   81

Joannard N   63

Johansen DN   42

Johansen D   52

Johansson U-B   120

Johnsen HR   80

Johnson S   18, 19, 117

Jones D   116

Jonsson KR   6, 7

Joubert N   73

Julián JA   71

Julia C   44

Jung J   116

Jurakić D   67

Jurakic D   11, 13

Juričan AB   82, 106

Jyväkorpi S   86

## K

Kaarsted T   42

Kaj M   8

Kallings LV   58

Kallings L   88, 109

Kalmari P   14

Kanso C   100

Karchynskaya V   90

Kari KE   33

Karim A-O   37

Kaseva K   98, 110

Kasper S   98

Kastelic K   89

Katapally TR   77

Kauhanen J   92

Kelleher CC   6, 7

Kelly E   82

Kelly L   22, 23, 24, 72

Kendrick D   85

Keskinen KE   85

Keskinen K   111

Keskinkilic B   8

Ketels M   46, 50, 61, 86, 115

Kim J   116

King A   84

Kirkegaard H   10

Kjellenberg K   78, 91

Klamroth S   101, 104

Klaus P   37

Klepp KI   47

Klepp K-I   62, 65

Klinker CD   80

Klump C   44

Kniely S   108

Knific T   82, 106

Knowles Z   17

Knudsen LS   40, 75

Koch S   62

Koelen M   93, 94

Kohler S   27, 37, 107

Kokko S   18, 57

Kokkorou M   47, 62, 65

Kolovou V   40, 69

Konieczna J   45

Kopčáková J   90

Korhonen N   63

Korpelainen R   57

Korshøj M   92, 93

Koutra V   83

Kovacs VA   5, 6, 8

Kozamernik J   47, 106

Krajewski P   22, 23

Kremlin W   66

Križan H   7

Kristjánsdóttir G   62

Kritz M   36

Kudlacek M   51

Kujundžic E   7

Kujundzic E   6

Kukko T   98

Kulmala J   110

Kurtzhals M   9, 10

## L

Léniz L   75

Lackinger C   12

Lahart IM   15, 17

Lakerveld J   22, 23, 24, 49

Lambe B   31, 106

Lana KD   33, 72

Lana S   97, 98

Lane A   72, 82

Langeard A   118

Langlois J   53

Larsen AH   79

Larsen R   62

Larsson K   120

Lazzaro A   103

Lebacq T   111

Lecomte E   53, 55

Lee L   34

Legrand K   53, 55

Lemonnier F   117

Lemos LFG   54

Lentillon-Kaestner V   47

Leon-Llamas JL   89

Leventi N   118

Levin O   83

Lhuisset L   52, 56, 68, 71

Lidin M   57

Lindert C van   49

Lion A   102

Lion A   74

Lippke S   117

Liston K   82

Ljungmann CK   80

Luiggi M   51

## M

Mäki P   7

Müller C   27, 28, 31

Müller I   73

MIzdrak A   96

Maaike H   46, 50, 86, 115

MacDonncha C   76

Mackintosh K   66, 78

Maddock J   76

Madrignac Y   103

Maenhout A   58

Maffiuletti N   118

Maillot J   94

Maindal HT   10

Makai A   95, 96

Malczyk S   65

Malek ME   39

Manneville F   53, 55, 98, 114

Mannix-McNamara P   76

Marec-Berard P   74

Margaritis I   112, 113

Maria O’Kane S   15, 17

Martinent G   45

Martins CL   54

Martins J   66

Marvalin S   74

Masiulis N   83

Masocco M   87, 97, 98

Massiera B   41, 48

Mathiassen SE   115

Mathisen JR   47

Matolić T   67

Matolic T   13

Matthews E   59

Matveeva M   88

Maury J   44

Mavoa S   45

Mazeas A   120

McBain T   37

McColl K   8

McDermott G   17

McEwan H   96

McGrath A   59

McMullen J   16

McNarry M   66, 78

McQuinn S   16

Mehtälä A   96

Meinadier E   94

Melan R   103

Melby PS   9, 10

Melkumova M   7

Menchawy IE   33

Mendes R   5, 6, 7, 8, 32, 38, 73

Mercer J   71

Meriaux-Scoffier S   47

Messing S   22, 23, 24, 49, 66

Mette K   93, 107

Meurisse B   64

Meyer J   117

Migal T   8

Milanović SM   6

Milanovic SM   5, 7

Milton K   38, 52, 66

Minardi V   87, 97, 98

Mino E   101, 104

Mocean F   102

Mongondry R   74

Moorlock S   34

Morawiak A   23

Morgan P   16

Morley S   78, 79

Morouço P   92, 99

Mortensen OS   92, 93, 107

Mota JG   54

Mota J   54, 66, 68, 97

Mota P   41

Moumjid-Ferdjaoui N   74

Moussay S   118

Mouton A   54, 102, 103

Muckelt PE   84

Mueller-Steck U   13, 14

Mulderij L   93, 94

Muller L   98, 114

Mullot M   64

Muncunill J   45

Murillo-Garcia A   89

Murillo-Pardo B   32

Murphy A   35

Murphy J   68, 99, 100

Murphy MH   15, 17

Murphy M   55, 66

Murphy N   31, 59, 106

Murray AD   84

Murtagh E   16, 50, 52

Mustafa SS   90

Myers D   116

## N

Nègre V   45

Naber I   101, 104

Narcis G   89

Nardone P   6, 7

Nauta J   81, 88

Ng K   55

Nicolson G   58

Nielsen G   9, 10, 80

Nielsen JV   40, 62, 75, 105

Niermann C   39

Noël-Racine A   48

Noel-Racine A   41

Nordenfelt A   57

Norman Å   39

Novak B   12

Nqweniso S   73

Ntoumanis N   81

Nurk E   6, 7

Nyberg G   39, 57, 78, 91

## O

O’Brien W   10

O'Keeffe B   76

O'Brian W   55

O'Kane M   15

O'Regan A   35, 91

O'Sulllivan N   35

Obreja G   8

Ocula C   103

Öhman H   86

Oida Y   61

Oja P   11

Okely AD   8

Okraszewska R   23

Olafsson AS   105

Oldenburg B   107

Oldridge-Turner K   47, 62, 65

Olesen LG   62

Olin S   57

Oliver E   48

Olsen MH   93

Omorou AY   55, 98, 114

Omorou A   44, 53

Onatsu T   61

Ooms L   18, 19

Orton E   85

Ossowsky MZ   83

Østergaard JN   10

Ostojic SM   6, 7

Overgaard AK   42

Owen K   38

## P

Pühse U   73

Padilla-Moledo C   39

Palwoski CS   36

Papiu A   61

Park M   116

Parkkari J   57

Patalay P   76

Patterson E   39

Pattison M   6

Paulsen L   31

Pawlowski CS   40, 75, 80

Pawlowski C   81

Pedersen MRL   109

Pedišić Ž   11, 25, 26, 27, 67, 89

Pedisic Z   11, 13

Pedroni C   111

Pehkonen-Elmi T   110

Perez-Bey A   39

Perol O   74

Peterkova V   6, 7

Peters M   117

Petitfrère Y   102, 103

Petrauskiene A   6, 7

Peytremann-Bridevaux I   33

Pfeifer K   27, 101, 104, 107

Pinard P   112

Pinedo A   7

Pinto R   99

Pischke CR   118

Pischke C   117

Platzer M   108

Plisson I   103

Podnar H   67

Podrekar N   89

Popova K   118

Popovic S   6

Popp J   27, 29

Portas E   4

Portegijs E   85, 111

Possenti V   87, 97, 98

Potdevin F   80

Poulsen VR   93, 107

Power D   31, 106

Præst C   67

Præstholm S   105

Prémusz V   95

Pratt M   25, 26

Preller L   13

Premusz V   96

Prins R   55

Prot B   103

Pudule I   6, 7

Puhan M   33, 72

Pulles I   49

Pykett J   84

## Q

Quarck G   118

Quennerstedt M   85

## R

Rütten A   27, 37

Racine AN   64, 113

Radman I   67

Radtke T   72

Raitakari OT   98

Raitakari O   110

Rakovac I   6, 7, 8

Ramigni M   87, 98

Rantanen T   85, 111

Rasmussen M   6

Rathmes G   7

Ratz T   117

Reiner M   108

Remacle M   54

Reuter CP   54

Rey O   51

Ribeiro J   50

Richards J   96

Richardson N   59

Riegler T   72

Rigby B   48

Rinta-Antila K   18

Rito AI   6, 7

Robin NSA   44

Robinson M   116

Rocha C   103

Rocha P   8, 20, 95, 96

Rocliffe P   76

Rodríguez-Rodríguez F   111

Romaguera D   45

Rompen J   102

Rossen J   120

Rostan F   117

Ružbarská B   90

Ruart S   71

Ruetten A   107

Ruf W   12

Ruiz M   45

Ruspini G   44

Rutter H   6, 7, 62

Rydberg JA   55

Ryde G   86

Ryom K   9, 10

## S

Sánchez-López S-L   66

Sääkslahti A   62, 63, 96

Sackou-Kouakou J-G   112

Saeid N   33, 113

Safsaf N   113

Salanave B   7

Salas-Salvadó J   45

Salavisa M   38

Salin K   54, 77, 110

Saloranta E   14

Samoilova J   88

Samuel D   83

Sanchez-Gomez J   89

Sandu P   61, 102

Sant'Angelo VF   7

Santos R   38

Saparkulovna IN   8

Šarabon N   89

Sarah F   49

Sarah T   49

Saucedo-Araujo RG   32

Savonen K   57

Sax A   102

Schindler K   6

Schipper M   21

Schipperijn J   31, 36, 81, 105

Schleberger S   118

Schmidt TB   105

Schmidt T   36, 40, 75

Scholl N   44

Schoonmade LJ   24

Schubert P   44

Scoffier-Mériaux S   94

Scott C   4, 5

Scott L   74

Seabra A   73

Šeflová I   91

Seghers J   14, 18, 36, 52

Segura-Diaz JM   32

Seil R   102

Selfe TK   116

Sellars P   71, 75

Semrau J   27, 29, 37, 107

Sevil-Serrano J   111

Seytor L-A   115

Sharp C   41, 66, 78

Shawley-Brzoska S   116

Shengelia L   6, 7

Shukurov S   8

Siersma V   92

Sigrist T   72

Silva CS   32, 38

Silva MN   32, 38

Simmonds P   8

Sing F   62

Singh A   55, 69

Sinnapah S   71

Škegro D   95

Sköld MB   107

Skovgaard T   51, 52, 62, 63, 67

Slot-Heijs J   55

Soares PF   42

Sofie K   31

Soini A   62

Solano AA   50

Sollerhed A-C   62

Sommer R   27

Soummer E   103

Speer A   39

Spielmanns M   72

Spinelli A   6, 7

Spiroski I   6, 7

Sschnitzler C   80

Staal MT   80

Staines A   16

van Stam W   49

Starc G   6, 8

Starck H   13, 14

Stathi A   34, 36, 84

Steffansson M   110

Stephanie W   44

Steurer-Stey C   33

Stojisavljević D   6

Stokes M   83, 84

Strandberg T   86

Strassmann A   33

Stratton G   78

Strehn A   12

Streicher H   39

Stuart AG   46

Sturua L   8

Suesse T   8

Sutton K   34

Swales B   86

Sweeney MR   16

## T

Tørslev MK   70

Takeda N   61

Tammelin T   63, 96, 98, 110

Tanrygulyyeva M   6, 7

Tcymbal A   66

Teehan M   72

Teixeira P   32, 38

Terragni L   59

Tezier B   117

Thérouanne P   45, 115, 119

Theisen D   102

Thiel A   27

Thogersen-Ntoumani C   36

Thommes L   102

Ticha L   6

Till M   27, 43

Tillander A   38

Titze S   11, 12, 108

Tlučáková L   90

Toft U   70

Tomás G-C   66

Toon H   36

Toussaint H   81

Toussaint J-F   64

Trinito MO   87, 98

Troelsen J   42, 79

Truwant L   98

Tuomola E-M   85, 111

Tuttner S   107

Tuunanen K   61

Tyler R   17

## U

van Uffelen J   36

Ullberg O   64

Urhausen A   102

Urtamo A   86

Usuopva Z   6, 7

Uusitalo A   57

## V

Vähä-Ypyä H   22

Väisänen D   109

Vašková M   90

Vale S   97

Valtonen M   57

Van Holland B   69, 70

Van Hoye A   18, 115, 117

Van Hulle D   58

van Biljon A   77

van Lenthe F   69

van Uffelen J   13, 14

van de Kop H   81

van den Berg L   65

van den Berg S   97, 114

van der Poel H   65

von Holdt K   117

Vanderlinden J   14, 36

Vardinghus-Nielsen H   70

Varela AR   25, 26

Vasankari T   20, 57

Vaughan L   76

Veiga OL   39

Veitch J   81

Verbaandert E   14

Veress R   12, 95, 96

Verger P   34

Verhoeff A   81

Verkooijen K   59, 93, 94, 105

Verloigne M   50

Vich G   45

Vilhjálmsson R   62

Villa-González E   32

Villafaina S   89

Villberg J   57

Vinet A   34

Vlad I   47, 62, 65

Vodenitcharova A   118

Voelcker-Rehage C   117

Volf K   22, 23, 24

Von Rosen P   120

Vuillemin A   41, 48, 64, 66, 112, 113, 115, 117

## W

Wärnberg J   45

Wagemakers A   59, 93, 94

Wagner P   39

Wahlström V   115

Walklett J   40

Wallin P   58

Wallmann-Sperlich B   31

Walsh L   76

Warner MB   84

Warner M   83

Weghuber D   6, 7

Weissenfels A   101, 104

Wen S   116

Wendel-Vos W   49, 65, 97, 114

Wernbacher T   108

Whitaker I   36

Whiting S   5, 6, 7, 8, 66

Whittaker A   86

Wickramasinghe K   5, 7

Wilcox S   116

Williams J   5, 6, 7

Willmot R   66

Wilson DA   84

Wirz M   119

Withall J   34, 36

Wood G   84

Woods CB   23, 24, 50

Woods C   22, 24, 35, 49, 55, 91

Wulff H   39

## Y

Yakimovich I   88

Yanakieva A   118

Yang X   98, 110

Yardim MS   7

Yardim N   6

Yuldashev R   8

## Z

Zacharides KK   14

Zaffuto C   44

Zamrazilová H   6, 7

Zaragoza J   68

Zhukova N   88

Ziegeldorf A   39

Žlender V   47

Zukowska J   22, 23, 24, 49

Zunquin G   68

Zutter D   119

